# Global and regional quality of care index for prostate cancer: an analysis from the Global Burden of Disease study 1990–2019

**DOI:** 10.1186/s13690-023-01087-2

**Published:** 2023-04-26

**Authors:** Ali Nowroozi, Shahin Roshani, Seyyed-Hadi Ghamari, Parnian Shobeiri, Mohsen Abbasi-Kangevari, Narges Ebrahimi, Negar Rezaei, Moein Yoosefi, Mohammad-Reza Malekpour, Mohammad-Mahdi Rashidi, Mana Moghimi, Erfan Amini, Alireza Namazi Shabestari, Bagher Larijani, Farshad Farzadfar

**Affiliations:** 1 Non-Communicable Diseases Research Center, Endocrinology and Metabolism Population Sciences Institute, Tehran University of Medical Sciences, Tehran, Iran; 2 Endocrinology and Metabolism Research Center, Endocrinology and Metabolism Clinical Sciences Institute, Tehran University of Medical Sciences, Tehran, Iran; 3 Uro-Oncology Research Center, Tehran University of Medical Sciences, Tehran, Iran; 4 Department of Geriatric Medicine, School of Medicine, Tehran University of Medical Sciences, Tehran, Iran

**Keywords:** Quality of care, Quality of care index, QCI, Prostate neoplasm, Global burden of disease

## Abstract

**Background:**

Prostate cancer (PCa) is one of the most prevalent cancers worldwide, with a significant burden on societies and healthcare providers. We aimed to develop a metric for PCa quality of care that could demonstrate the disease’s status in different countries and regions (e.g., socio-demographic index (SDI) quintiles) and assist in improving healthcare policies.

**Methods:**

Basic burden of disease indicators for various regions and age-groups were retrieved from Global Burden of Disease Study 1990–2019, which then were used to calculate four secondary indices: mortality to incidence ratio, DALYs to prevalence ratio, prevalence to incidence ratio, and YLLs to YLDs ratio. These four indices were combined through a principal component analysis (PCA), producing the quality of care index (QCI).

**Results:**

PCa’s age-standardized incidence rate increased from 34.1 in 1990 to 38.6 in 2019, while the age-standardized death rate decreased in the same period (18.1 to 15.3). From 1990 to 2019, global QCI increased from 74 to 84. Developed regions (high SDI) had the highest PCa QCIs in 2019 (95.99), while the lowest QCIs belonged to low SDI countries (28.67), mainly from Africa. QCI peaked in age groups 50 to 54, 55 to 59, or 65 to 69, depending on the socio-demographic index.

**Conclusions:**

Global PCa QCI stands at a relatively high value (84 in 2019). Low SDI countries are affected the most by PCa, mainly due to the lack of effective preventive and treatment methods in those regions. In many developed countries, QCI decreased or stopped rising after recommendations against routine PCa screening in the 2010–2012 period, highlighting the role of screening in reducing PCa burden.

**Supplementary Information:**

The online version contains supplementary material available at 10.1186/s13690-023-01087-2.

## Background

Prostate cancer (PCa) is the second most incident cancer among men worldwide, affecting more than 1.4 million cases annually [1]. From 1990 to 2017, the age-standardized incidence rate (ASIR) of PCa had increased from 30.5 to 37.9 per 100,000, globally [2]. In the same period, although the total number of PCa related deaths had nearly doubled, the age-standardized death rate (ASDR) had dropped by 2.1 percent [2]. High socio-demographic index (SDI) regions experienced sharp declines in PCa ASDR from 1990 to 2017, while regions with lower SDIs showed slighter decreases or even increases of ASDR in the same period [2].

The World Health Organization defines quality of care (QoC) as the degree to which health services increase the chance of desired health outcomes [3]. The American Society of Clinical Oncology has, in a statement, highlighted some of the disparities present in cancer care in the United States [4] and has since provided guidance on how to reduce these discrepancies and overcome the challenges [5, 6]. Despite all efforts, healthcare inequalities exist even in high-income countries, let alone countries with lower incomes. Countries with better QoC, if identified, could serve as role models for other nations.

Several studies have developed indices to measure the quality of patient care [7, 8]. However, a reliable numerical QoC indicator that could be used for all diseases is yet to be defined. In this study, we present the newly introduced “quality of care index” (QCI) [9] and implement it on PCa data obtained from the Global Burden of Disease (GBD) study 1990–2019 [10] in order to achieve a global overview on PCa QoC and potentially assist healthcare experts and policy-makers in improving it.

## Methods

### Data sources

Data used in our analyses were acquired from the Institute for Health Metrics and Evaluation GBD database [11] from 1990 to 2019, using the disease code B.1.18 for PCa, which mapped International Classification of Diseases codes C61-61.9, D07.5, D29.1, D40.0, Z12.5, Z80.42, and Z85.46 [12].

### The quality of care index

The QCI, as also described by others [9, 13-23], is an index ranging from 0 to 100 which is directly correlated to the QoC or early detection of a disease in a given region. Its method of calculation is described in the following section.

### Calculation of the QCI

#### Primary variables

The primary variables extracted from the GBD data were prevalence, incidence, mortality, disability adjusted life years (DALYs), years of life lost (YLLs), and years lived with disability (YLDs). We then used these indices to calculate secondary variables, and consequently, the QCI.

#### Secondary variables

For each location (country, region, etc.), four indices (secondary variables) were calculated from the obtained data (primary variables):

1 $$\mathrm{DALYs\;to\;Prevalence\;Ratio}= \frac{\mathrm{DALYs}}{\mathrm{Prevalence}}$$

2 $$\mathrm{YLLs\;to\;YLDs\;Ratio}=\frac{\mathrm{YLL}}{\mathrm{YLD}}$$

3 $$\mathrm{Prevalence\;to\;Incidence\;Ratio}=\frac{\mathrm{Prevalence}}{\mathrm{Incidence}}$$

4 $$\mathrm{Mortality\;to\;Incidence\;Ratio }(\mathrm{MIR})=\frac{\mathrm{Mortality}}{\mathrm{Incidence}}$$

These indices are carefully selected by the scientists of our institute and each represent an aspect of quality of care. In an ideal quality of care state, patient identification is maximal (high incidence) and patients are cured and become disease free in a relatively short time after diagnosis (low prevalence) (index 3). Moreover, burden and mortality rates are kept at their minimums (indinces 1 and 4), and diseases tend to be chronic rather than fatal, if no definitive treatment is available (index 2). We combined these four indices into a single index using principal component analysis. This statistical method simplifies multi-dimensional large datasets into few principal components while preserving variance of the data to a great degree [24]. QCI was defined as the first principal component of the analysis, which has the maximum data preservation [9]. Details of the mathematical calculations are available in the QCI protocol published by Mohammadi et al. [9].

### Target regions

Our analyses were focused on global QCI including 204 countries and five SDI quintiles. The SDI is a number between 0 and 1 generated from combining income per capita (direct correlation), educational attainment (direct correlation), and total fertility rate (opposite correlation), which is indicative of a region’s socio-demographic development [25]. Accordingly, geographies are categorized into low, low-middle, middle, high-middle, and high SDI groups (Supplementary Table S1).

### Age disparity

To demonstrate PCa QoC for different ages, we defined 14 age groups, encompassing five-year intervals (20 to 24, 25 to 29, …, 80 to 84, 85 +). In instances where the age-group is not mentioned, we reported the age-standardized QCI values.

### Quality of care index validation

We validated the QCI by performing a mixed-effect regression analysis with two indices developed by the Institute for Health Metrics and Evaluation used for measuring healthcare quality in various diseases: the Healthcare Access and Quality Index [26] and the Universal Health Coverage effective health coverage index [27]. QCI was used as the dependent variable, and inpatient healthcare utilization, outpatient healthcare utilization, mortalities of all risk factors, and prevalence were designated as independent variables. Countries effect was considered as a random effect. Results indicated that QCI was highly correlated with the two indices, with Pearson correlation coefficients of 0.78 for Healthcare Access and Quality Index and 0.74 for the Universal Health Coverage index.

### Statistical analyses

Primary indices were reported with 95% uncertainty intervals (UI). Changes were considered significant when the UIs did not overlap. All statistical analyses were performed using R statistical packages v4.0.4 (http://www.r-project.org/, RRID = SRC_001905).

## Results

### Burden of disease indices

Prostate cancer’s ASIR increased from 1990 to 2019 (34.1 (95% UI 26.8 – 39.6) to 38.6 (33.6 – 49.8)), while its ASDR decreased in the same period (18.1 (14.7 – 21.2) to 15.3 (13.0 – 18.6)) (supplementary table S2). The age-standardized rate (ASR) of DALYs also decreased (286.3 (232.8 – 326.2) to 244.1 (211.8 – 297.7)), along with YLLs’ share of DALYs (92.3% vs 89.6% for 1990 and 2019, respectively).

Prostate cancer became significantly more prevalent in all SDI quintiles from 1990 to 2019. In this period, the ASIR and the ASR of YLDs significantly increased in low-middle and middle SDI countries, while no significant changes were observed in the ASRs of DALYs and YLLs in any of the SDI regions.

### Quality of care index

**Fig. 1:**
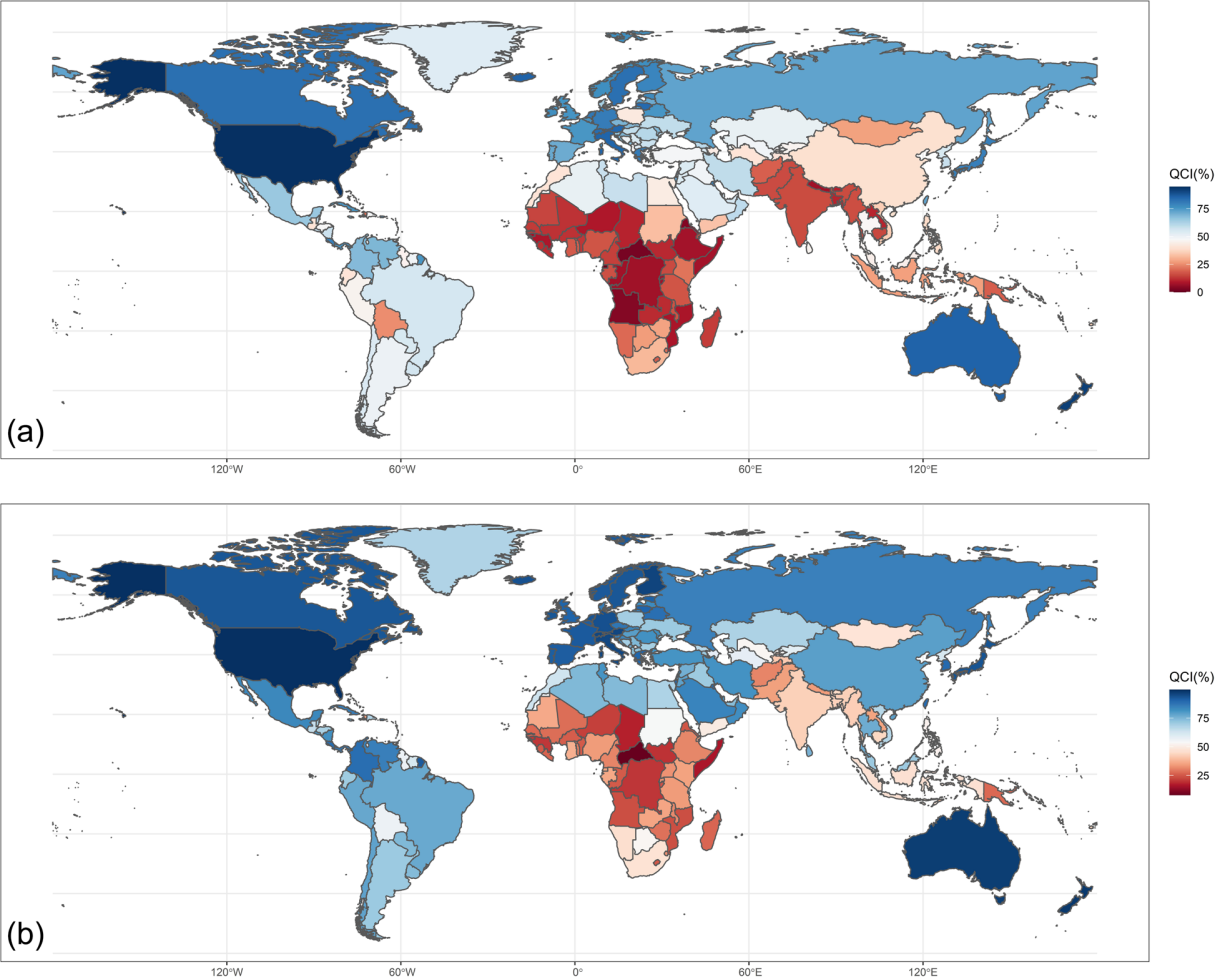
Geographical distribution of age-standardized QCI (**a**) 1990, (**b**) 2019

Global QCI for PCa increased from 74.0 in 1990 to 84.0 in 2019. In 2019, the United States of America (QCI = 99.7) and Central African Republic (QCI = 8.0) had the highest and lowest QCIs, respectively (Fig. 1b, Supplementary Figure S1, Supplementary Table S3). Minimum and maximum QCIs of 2019 were both higher than those of 1990 (Fig. 1, Tables 1 and 2). In fact, all countries experienced growths in QCI during the 30-year period, ranging from + 0.4 (Zimbabwe) to + 41.6 (Maldives) (Supplementary Tables S4 and S5). The United States has consistently led in PCa QoC from 1990 (QCI = 94.7) to 2019 (QCI = 99.7). On the other end of the spectrum, countries with the lowest QCIs have almost exclusively comprised African countries, with the Central African Republic having the lowest QCI since 1994.

**Table 1:** Countries with highest QCIs

Rank	1990	1990 QCI	2019	2019 QCI	Rank change	QCI change
1	USA	94.67	USA	99.71	-	+ 5.04
2	New Zealand	91.72	New Zealand	98.04	-	+ 6.32
3	Australia	85.69	Australia	97.44	-	+ 11.75
4	Iceland	85.63	Finland	96.14	+ 17	+ 17.49
5	Italy	85.48	Austria	95.77	+ 1	+ 10.74
6	Austria	85.03	Italy	95.56	-1	+ 10.08
7	Lithuania	84.39	Switzerland	95.51	+ 2	+ 11.72
8	Monaco	83.80	Germany	94.20	+ 9	+ 13.84
9	Switzerland	83.79	Malta	93.91	+ 6	+ 13.20
10	Canada	82.99	Iceland	93.90	-6	+ 8.28

**Table 2:** Countries with lowest QCIs

Rank	1990	1990 QCI	2019	2019 QCI	Rank change	QCI change
204	Equatorial Guinea	0	Central African Republic	7.99	-1	+ 5.83
203	Central African Republic	2.16	Somalia	16.4	-10	+ 8.72
202	Eritrea	3.68	Chad	17.84	-15	+ 7.33
201	Angola	3.73	South Sudan	19.9	-17	+ 8.92
200	Rwanda	6.96	Guinea-Bissau	20.23	-5	+ 12.69
199	Nepal	7.2	Democratic Republic of the Congo	20.29	-1	+ 13.09
198	Democratic Republic of the Congo	7.21	Guinea	20.46	-7	+ 11.88
197	Burundi	7.22	Niger	21.61	-7	+ 12.61
196	Congo	7.35	Mozambique	23.78	-4	+ 15.23
195	Guinea-Bissau	7.53	Angola	23.81	+ 6	+ 20.08

Higher SDIs had higher QCIs in 1990 and 2019, as expected (Supplementary Figure S2). Although low SDI countries had the highest growth rate from 1990 to 2019 (almost 2.5-fold increase from 12.3 to 28.7), high SDI regions have been the most successful in approaching the optimal QoC which is a QCI rating of 100 (from 87.2 to 96.0, 68.8% closer to 100).

Among the seven GBD super-regions, 2019 QCI was highest in High-Income (95.6), Latin America and Caribbean (80.1), and Central Europe, Eastern Europe, and Central Asia (80.0) regions, followed by North Africa and Middle East (76.3), Southeast Asia, East Asia, and Oceania (75.1), South Asia (42.1), and Sub-Saharan Africa (33.0) (Supplementary Figure S3).

**Fig. 2:**
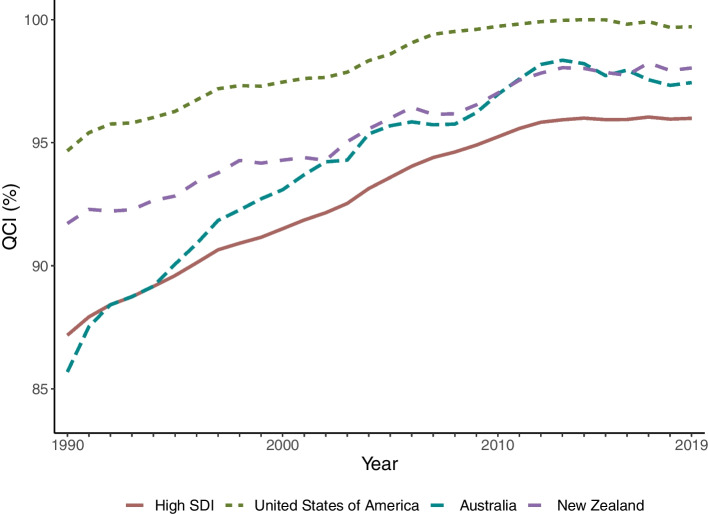
Trend of age-standardized QCI in the top three countries and high SDI region

Figure 2 shows QCI trend throughout the years in the USA, New Zealand, Australia (the top three countries), and high SDI regions, where QCI peaked in 2014 (100), 2017 (98.2), 2013 (98.35), and 2017 (96.04).

### Quality of care index age trend

**Fig. 3:**
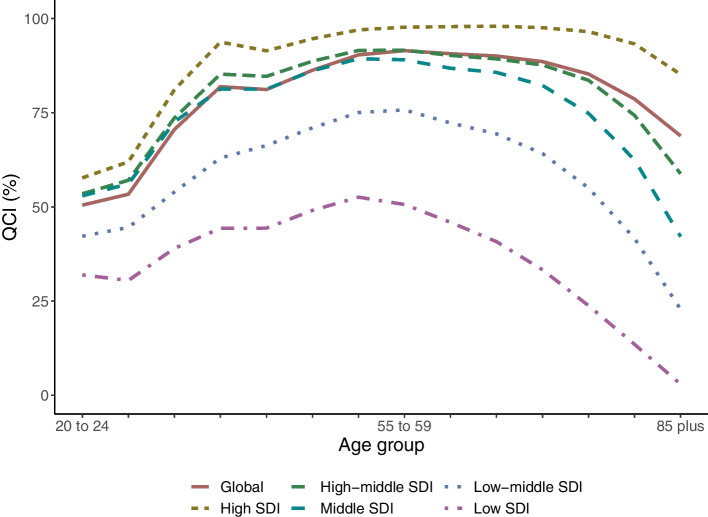
Distribution of QCI by age group and SDI in 2019

The QCI peaked at three age groups across different SDI quintiles (Fig. 3): 50 to 54 (middle (QCI = 89.3) and low (52.6) SDIs), 55 to 59 (globally (91.5), and high-middle (91.6) and low-middle SDIs (75.8)), and 65 to 69 (high SDI (98.0)). On the other hand, the lowest QCIs were observed either in the youngest of patients (i.e., ages 20 to 24, globally (50.5), and in high (57.7) and high-middle (53.5) SDI quintiles), or in the other end of the spectrum (i.e., ages above 85, middle (42.1), low-middle (22.8) and low (2.9) SDs).

## Discussion

Globally, ASIR and ASR of YLDs of PCa have increased from 1990 to 2019, while ASDR and ASR of DALYs and YLLs have decreased. Furthermore, the rate of DALYs has decreased in high and high-middle SDI regions and increased in regions with middle, low-middle, and low SDIs. Prostate cancer QoC has increased globally and in every SDI region over the 30-year period.

The increase in PCa incidence in the early 90 s could largely be attributed to the introduction of PSA as a screening method [28]. The trend later leveled off, most likely due to overall healthcare improvements around the globe and the success of preventive measures including early detection. Difference in PCa screening, diagnosis, and management is a logical explanation for the QCI gap between high and low SDI regions [29]. Furthermore, low-middle and middle SDIs were the only quintiles with significant ASIR increases from 1990 to 2019. This might be due to the persistence of ineffective screening in low SDI countries and successful control and prevention of the disease in high-middle and high SDI regions. Reduced PSA testing as recommended by the newest guidelines could have also contributed to reduced PCa incdince in high income countries [2, 30].

QoC indicators enables regulatory organizations to develop new policies and evaluate the existing ones more accurately. The QCI has unique qualities compared with other QoC indicators [26, 27, 31]: it is easily calculated from basic epidemiological indices, could be applied to all diseases [13-15] and demographic classifications, and most importantly, produces a single numerical index that is objectively interpreted and is capable of being compared between groups.

Global PCa QCI has increased by nearly 14% since 1990. Regions with higher SDIs and high-income regions have had higher QCIs throughout the years, and all of the countries on top of the QCI ranking are highly developed nations. On the other hand, the bottom of the list mainly comprises African countries with low SDIs. This is unsurprising, given the heavy costs of PCa on economies and individuals [32-35].

Nevertheless, our findings indicate that sub-Saharan countries with relatively high SDIs (such as South Africa with SDI = 0.68) have QCIs well below the global average (South Africa 2019 QCI = 47.1), similar to other nations of the sub-Saharan Africa. This highlights the role of contributing factors other than income and socio-demographic development. These include lack of information about the disease [36], screening policies [36], and local guidelines optimized for the region [35]. Ethnicity also plays a role; for instance, African-American ethnicity is a known risk factor of PCa and the disease’s burden is heavy in countries where many people are from African origins [2, 37].

The effect of prostate-specific antigen (PSA)-based screening on QCI is evident in many of our findings. QCI surged in many countries in the mid 1990s, which is most likely due to introduction of PSA and its subsequent approval by the US Food and Drug Administration as a screening tool for prostate cancer [38]. However, in 2009, the results of two large clinical trials [39, 40] questioned the benefits of PSA screening, and consequently, policymakers revisited their stance on PCa screening. The updated American Cancer Society guidelines for PCa screening in 2010 [41] emphasized on a more patient-oriented approach rather than the strong recommendations in favor of PSA-based screening presented in the previous 2001 guidelines [42]. Later in 2012, the United States Preventive Services Task Force (USPSTF) released a statement recommending against PSA screening (grade D), for all age groups [43]. These alterations led to many unfavorable outcomes. According to two studies in the US [44] and Australia [45], the 2012 statement resulted in a higher risk of harboring a more aggressive disease upon diagnosis. In countries with high PCa QCIs (e.g., the USA, New Zealand, and Australia) and high SDI regions, ASRs of mortality, DALYs, YLDs, and YLLs, which had constantly been decreasing, have started to rise in the past few years, resulting in the cessation of the upward trend of QCI in these regions. Finally, in 2018, USPSTF changed its stance on PSA-based screening from “discouraged in all age groups” (grade D) to “individualized recommendation in ages 55 to 69” (grade C) [46], which is similar to American Urological Association’s guidelines [47]. It is a matter of time to see whether this change affects PCa burden and quality of care or not. Our results are in favor of PSA screening as QCI had increased whenever policies were in favor of screening and decreased when screenings were limited, although PSA testing could lead to overdiagnosis and overtreatment, and consequently, therapy complications such as incontinence and erectile dysfunction. Further studies and trials are required to clarify the benefits or disadvantages of PSA-based PCa screening. In addition, novel biomarkers are constantly being developed, which might have better diagnostic accuracy than PSA and reduce the adverse effects of overtreatment [48, 49].

Age disparity analyses revealed that QCI was lowest in both age extremities and peaked in middle-aged patients (ages 50 to 69 years) in 2019. Older PCa patients are more likely to have a more aggressive disease [50, 51] and are at higher chance of biochemical recurrence after treatment [51]. Also, younger PCa patients with advanced disease are at higher risk of poor outcomes [52, 53], although disease mortality in young patients regardless of their disease stage is of controversy [52-55] and unlike the elderly [50], no definite marker or genetic component for severe disease has been identified in these patients [54, 56]. One explanation for these findings could be the fact that guidelines do not recommend offering PCa testing to men > 70 years old and/or those with life expectancies < 10 years, and therefore, these patients are missed in the preliminary asymptomatic stages. The same is true for younger patients as PSA testing is only recommended to be discussed with patients above 40, 45, 50 or 55 years of age (depending on risk factors and the guideline) [41, 46, 47, 57].

Analyzing healthcare systems and practice guidelines of regions with continuously rising QCIs could guide experts in improving current protocols and guidelines. One outlier is Turkey, in which an abrupt increase of QCI is seen in 2003 and has persisted through 2019. The healthcare reform of Turkey (2003–2010) [58] is undoubtedly one of the main factors behind this improvement and could act as a role model for policymakers in other nations.

Our study is the first to evaluate quality of care of prostate cancer in a quantifiable method and developed the QCI at global and regional levels. The newly introduced QCI has valubale implications in public health, such as helping experts identify and understand disparities of quality of care between different groups and regions, and guiding organizations and societies through establishing healthcare policies to overcome PCa care challenges. Nonetheless, our study bears several limitations. Our data are acquired from the GBD database, and therefore, any limitation applying to it also applies to our study. Since GBD estimates its measures from various sources and using complex statistical methods, the results are subject to uncertainties. In addition, there are no classifications based on demographic characteristics such as race and risk factors. Furthermore, utility of a calculated QCI is limited to that disease and QCIs of different diseases could not be compared to each other. Moreover, we were unable to calculate uncertainty intervals for all calculated indices due to limited computational resources. Currently available literature implementing the QCI, to the best of our knowledge, are limited to our institution and additional studies by other research groups are required to further evaluate the QCI.

## Conclusions

Prostate cancer QoC has improved in the 1990–2019 period, as measured by the QCI. Regions with higher SDIs have better QoC, as demonstrated by QCI, which is mainly due to more effective healthcare systems and early initiation of screening. Factors other than socio-demographic development also contribute to PCa QoC, such as patient education, the healthcare system, and the presence of practical guidelines. Although PSA screening is a matter of debate and is nowadays not as strongly recommended as before, our study shows that QCI has decreased since the introduction of updated guidelines, including statements against routine PSA screening. We also compared QCI in different age groups and found that patients with either very low or very high age experience lower quality of care, which could be attributed to factors such as the more aggressive nature of the disease, undertreatment, and lack of PSA screening in these age groups. The results of our study could be used to identify disparities in healthcare and assist public health experts in taking appropriate actions in improving PCa QoC, such as modifying screening guidelines and resource allocation.

## Supplementary Information

**Additional file 1: Supplementary Figure S1.** Ranking of age-standardized QCI by country in 2019.

**Additional file 2: Supplementary Figure S2.** Age-standardized QCI by socio-demographic index.

**Additional file 3: Supplementary Figure S3.** QCIs for the seven GBD super-regions in 1990 and 2019.

**Additional file 4: Supplementary Table S1.** SDI values for different regions.

**Additional file 5: Supplementary Table S2.** Global and SDI-based regional epidemiologic indices of prostate cancer.

**Additional file 6: Supplementary Table S3.** QCIs of all countries from 1990 to 2019.

**Additional file 7: Supplementary Table S4.** Countries with highest QCI changes.

**Additional file 8: Supplementary Table S5.** Countries with lowest QCI changes.

## Data Availability

All data were gathered from the GBD results tool (http://ghdx.healthdata.org/gbd-results-tool) and GBD compare (https://vizhub.healthdata.org/gbd-compare/). Instruction for calculating the QCI are available in the protocol by Mohammadi et al. [9].

## Abbreviations

PCa: Prostate cancer
ASIR: Age-standardized incidence rate
ASDR: Age-standardized death rate
SDI: Socio-demographic index
QoC: Quality of care
QCI: Quality of care index
GBD: Global Burden of Disease
DALYs: Disability adjusted life years
YLLs: Years of life lost
YLD: Years lived with disability
MIR: Mortality to incidence ratio
UI: Uncertainty interval
ASR: Age-standardized rate
PSA: Prostate-specific antigen
USPSTF: United States Preventive Services Task Force
