# Supplementary material for: Global and regional quality of care index for prostate cancer: an analysis from the Global Burden of Disease study 1990–2019
Source: Arch Public Health. 2023 Apr 26;81:70. doi: 10.1186/s13690-023-01087-2 (PMC10131390; doi:10.1186/s13690-023-01087-2)
Supplement: Supplementary file 1 — Additional file 1: Supplementary Figure S1. Ranking of age-standardized QCI by country in 2019. [file 13690_2023_1087_MOESM1_ESM.pdf]

Country	QCI	Country	QCI	Country	QCI	Country	QCI	Country	QCI
United States of America	99.71	Slovenia	84.92	Saint Lucia	71.70	Philippines	51.95	Mali	28.10
New Zealand	98.04	Russian Federation	84.92	Trinidad and Tobago	71.07	Tonga	50.42	Senegal	27.41
Australia	97.44	Saudi Arabia	84.43	Ukraine	70.88	Yemen	49.79	Papua New Guinea	27.32
Finland	96.14	Slovakia	83.93	Mauritius	70.82	Mongolia	47.52	Madagascar	26.97
Austria	95.77	Mexico	83.65	Argentina	70.75	South Africa	47.15	Liberia	26.84
Italy	95.56	Croatia	83.49	Armenia	70.65	Indonesia	46.80	Kiribati	26.26
Switzerland	95.51	Latvia	82.91	Malaysia	70.60	Namibia	45.93	Burkina Faso	25.86
Germany	94.20	Oman	82.28	Iraq	70.45	Tuvalu	45.05	Benin	25.56
Malta	93.91	Republic of Moldova	82.16	Ecuador	70.19	Bhutan	44.71	Congo	25.54
Iceland	93.90	Nicaragua	81.99	Poland	69.92	Cambodia	44.32	Sierra Leone	25.42
Japan	93.57	Romania	81.68	Palestine	69.61	Fiji	43.24	Côte d'Ivoire	25.11
Netherlands	93.26	Iran (Islamic Republic of)	81.48	Bosnia and Herzegovina	69.61	Bangladesh	43.23	Lesotho	24.97
Ireland	93.10	Turkey	81.22	Bulgaria	69.22	India	43.02	Eritrea	24.80
Canada	93.05	Tunisia	81.12	Honduras	68.71	Micronesia (Federated States of)	42.68	Burundi	24.15
Sweden	92.96	El Salvador	80.98	Belize	68.46	Myanmar	42.55	Angola	23.81
Andorra	92.68	Qatar	80.46	Kazakhstan	68.43	Tajikistan	41.77	Mozambique	23.78
Luxembourg	92.62	Barbados	79.70	Egypt	68.18	Solomon Islands	40.90	Niger	21.61
Norway	92.61	Hungary	79.25	Guam	68.05	Sao Tome and Principe	39.97	Guinea	20.46
France	92.48	China	79.17	Greenland	67.93	Timor-Leste	38.89	Democratic Republic of the Congo	20.29
Cyprus	92.43	Jordan	78.66	Dominican Republic	67.74	Ghana	37.44	Guinea-Bissau	20.23
Spain	92.01	Bahrain	78.59	Viet Nam	67.07	Djibouti	36.44	South Sudan	19.90
Belgium	91.94	Chile	78.50	Palau	67.01	Mauritania	36.27	Chad	17.84
Portugal	91.68	Peru	77.59	Georgia	66.80	Gabon	36.21	Somalia	16.40
Estonia	91.26	Saint Kitts and Nevis	77.33	Niue	66.40	Zambia	35.75	Central African Republic	7.99
Singapore	91.17	Brazil	77.23	Seychelles	66.25	Equatorial Guinea	35.67		
San Marino	91.07	United States Virgin Islands	77.06	Brunei Darussalam	65.69	Lao People's Democratic Republic	35.31		
United Kingdom	90.90	Thailand	76.76	Guatemala	64.90	Kenya	35.25		
Lithuania	90.54	Montenegro	76.45	Saint Vincent and the Grenadines	64.19	Pakistan	34.90		
Greece	90.38	Syrian Arab Republic	76.09	Dominica	63.85	Haiti	34.66		
Monaco	90.25	Maldives	76.00	Morocco	63.20	Nepal	34.40		
Bermuda	90.08	Antigua and Barbuda	75.93	Suriname	61.57	United Republic of Tanzania	34.31		
Israel	90.06	Sri Lanka	75.57	Democratic People's Republic of Korea	61.54	Nigeria	34.08		
Republic of Korea	89.62	Jamaica	75.56	Kyrgyzstan	58.63	Rwanda	33.50		
Taiwan (Province of China)	89.51	Serbia	74.57	Turkmenistan	58.26	Marshall Islands	33.45		
Costa Rica	89.16	Algeria	74.56	Azerbaijan	58.21	Uganda	33.12		
Puerto Rico	89.00	Paraguay	74.48	Bolivia (Plurinational State of)	56.86	Eswatini	32.67		
Colombia	88.96	Libya	74.12	Guyana	56.83	Malawi	31.80		
Kuwait	88.81	Uruguay	73.99	Cabo Verde	55.67	Ethiopia	31.39		
Belarus	88.57	Northern Mariana Islands	73.81	Tokelau	55.22	Cameroon	31.20		
Czechia	87.19	United Arab Emirates	73.55	Samoa	55.18	Afghanistan	31.14		
Panama	86.89	Albania	73.29	American Samoa	54.52	Comoros	31.00		
Cuba	85.81	Cook Islands	72.97	Sudan	54.09	Togo	30.09		
Denmark	85.51	Bahamas	72.65	Nauru	53.33	Gambia	29.44		
Lebanon	85.51	North Macedonia	72.05	Botswana	53.13	Vanuatu	29.35		
Venezuela (Bolivarian Republic of)	85.16	Grenada	71.75	Uzbekistan	53.04	Zimbabwe	28.53		
