# Supplementary figures and images for: Global and regional quality of care index for prostate cancer: an analysis from the Global Burden of Disease study 1990–2019
Source: Arch Public Health. 2023 Apr 26;81:70. doi: 10.1186/s13690-023-01087-2 (PMC10131390; doi:10.1186/s13690-023-01087-2)

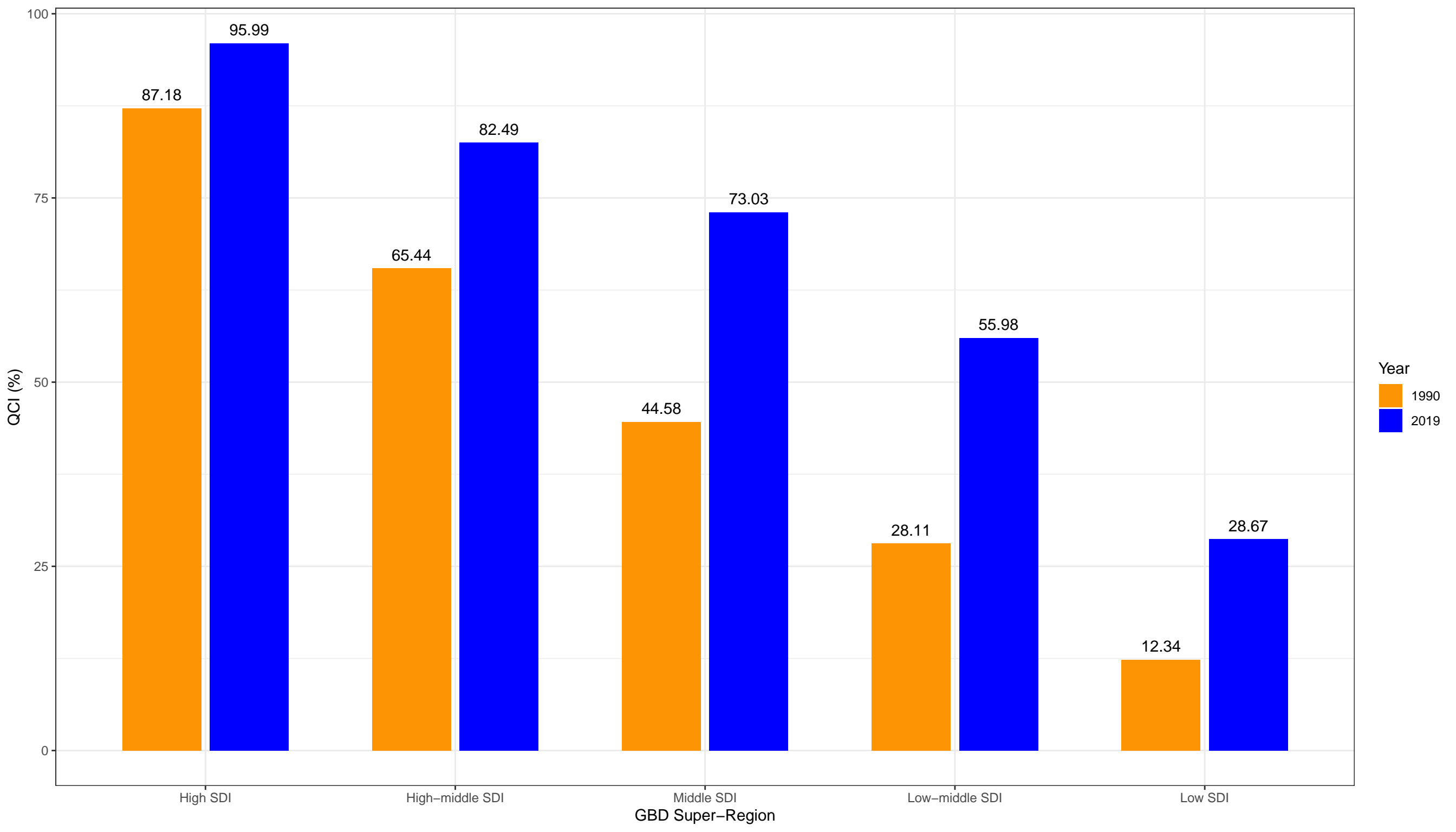

Supplement: Supplementary file 2 — Additional file 2: Supplementary Figure S2. Age-standardized QCI by socio-demographic index. [file 13690_2023_1087_MOESM2_ESM.pdf]

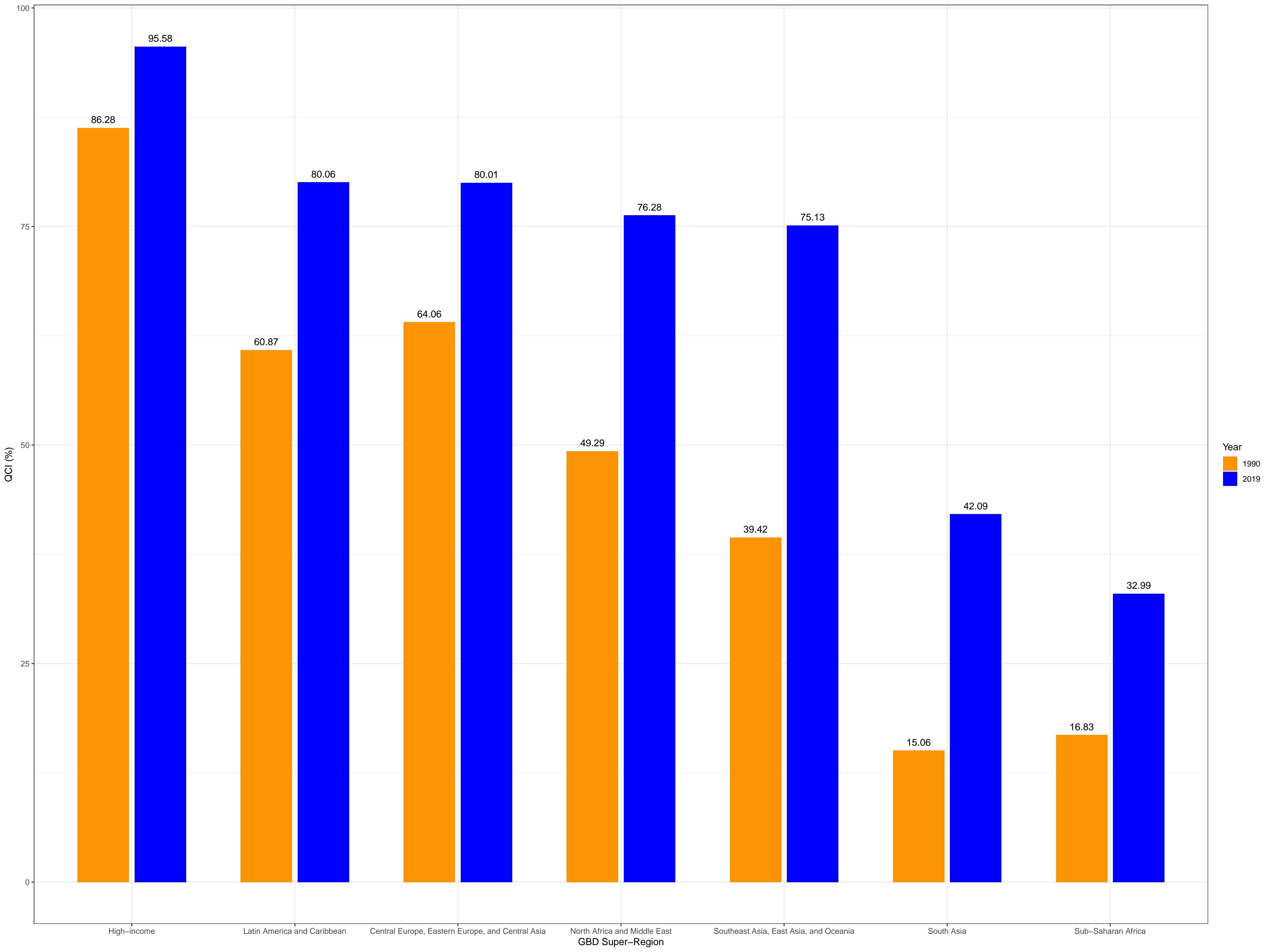

Supplement: Supplementary file 3 — Additional file 3: Supplementary Figure S3. QCIs for the seven GBD super-regions in 1990 and 2019. [file 13690_2023_1087_MOESM3_ESM.pdf]
