# Supplementary material for: Global and regional quality of care index for prostate cancer: an analysis from the Global Burden of Disease study 1990–2019
Source: Arch Public Health. 2023 Apr 26;81:70. doi: 10.1186/s13690-023-01087-2 (PMC10131390; doi:10.1186/s13690-023-01087-2)
Supplement: Supplementary file 5 — Additional file 5: Supplementary Table S2. Global and SDI-based regional epidemiologic indices of prostate cancer. [file 13690_2023_1087_MOESM5_ESM.pdf]

Region	Year	Prevalence		Incidence		Deaths		DALYs		YLDs		YLLs	
		Number	Rate	Number	Rate	Number	Rate	Number	Rate	Number	Rate	Number	Rate
Global	1990 (95% UI)	3651763 (2880852 to 4216030)	223.3 (177.3 to 254.4)	524110 (409133 to 613006)	34.1 (26.8 to 39.6)	232999 (191398 to 268882)	18.1 (14.7 to 21.2)	4360506 (3528030 to 4951007)	286.3 (232.8 to 326.2)	334557 (230361 to 441154)	21.1 (14.5 to 27.4)	4025949 (3267352 to 4549667)	265.2 (218.1 to 304.6)
	2019 (95% UI)	10812979 (9538731 to 13977819)	286.8 (253.3 to 368.7)	1410452 (1227900 to 1825766)	38.6 (33.6 to 49.8)	486836 (420498 to 593689)	15.3 (13.0 to 18.6)	8644870 (7548021 to 10559866)	244.1 (211.8 to 297.7)	902697 (644209 to 1239866)	24.3 (17.4 to 33.4)	7742173 (6714725 to 9327541)	219.7 (189.8 to 265.7)
	Significant?	*		*		*		*		*		*	
Low SDI	1990 (95% UI)	49719 (38292 to 58136)	46.2 (36.1 to 53.6)	14667 (10948 to 17314)	16.2 (12.0 to 19.2)	14405 (10567 to 17042)	17.4 (12.8 to 20.6)	291434 (213455 to 346280)	290.9 (213.6 to 344.7)	6081 (4104 to 8289)	6.1 (4.1 to 8.3)	285352 (209169 to 338933)	284.8 (209 to 337.2)
	2019 (95% UI)	174620 (135461 to 205746)	74.5 (58.2 to 87.5)	35447 (27190 to 41728)	17.7 (13.6 to 20.7)	35219 (25881 to 41598)	19.5 (14.2 to 22.9)	681262 (501507 to 806489)	320.4 (235.1 to 378.2)	18452 (12503 to 25482)	8.5 (5.8 to 11.6)	662810 (488220 to 786758)	311.9 (229.7 to 368.7)

Region	Year	Prevalence		Incidence		Deaths		DALYs		YLDs		YLLs	
		Number	Rate	Number	Rate	Number	Rate	Number	Rate	Number	Rate	Number	Rate
	Significant?	*	*	*		*		*		*		*	
Low-middle SDI	1990 (95% UI)	117088 (101629 to 139416)	43.7 (38.2 to 52.4)	27668 (23401 to 32999)	12.4 (10.4 to 15.0)	24287 (19955 to 28874)	12.3 (10.1 to 14.9)	467412 (384211 to 546827)	193.1 (159.1 to 229.1)	12450 (9139 to 16279)	5.0 (3.7 to 6.6)	454962 (372866 to 533780)	188.1 (154.3 to 223.1)
	2019 (95% UI)	601917 (515664 to 701809)	95.6 (82.3 to 111.2)	100389 (86423 to 117896)	17.8 (15.3 to 21.0)	66632 (56325 to 78818)	13.5 (11.4 to 16.0)	1215480 (1018740 to 1427257)	212.9 (179.3 to 250.8)	55219 (40494 to 72050)	9.2 (6.8 to 12.1)	1160261 (975483 to 1358506)	203.7 (171.7 to 240.6)
	Significant?	*	*	*	*	*		*		*	*	*	
Middle SDI	1990 (95% UI)	288383 (247218 to 331114)	64.7 (56.2 to 74.6)	53502 (45509 to 61366)	14.5 (12.5 to 16.9)	39007 (32789 to 44649)	12.6 (10.8 to 14.8)	743444 (618726 to 838434)	191.2 (160.8 to 220.2)	27330 (19883 to 35704)	6.7 (4.9 to 8.6)	716114 (595715 to 807129)	184.5 (154.6 to 211.7)
	2019 (95% UI)	1715574 (1491963	148.7 (129.9	227646 (195373	21.7 (18.6	112213 (94003	12.8 (10.6	2026389 (1710329	197.2 (165.6	147218 (106339	13.4 (9.7	1879172 (1584713	183.9 (154.2

Region	Year	Prevalence		Incidence		Deaths		DALYs		YLDs		YLLs	
		Number	Rate	Number	Rate	Number	Rate	Number	Rate	Number	Rate	Number	Rate
		to 2047469)	to 177.3)	to 271989)	to 25.8)	to 133289)	to 15.2)	to 2399588)	to 234.8)	to 195471)	to 17.7)	to 2223715)	to 218.7)
	Significant?	*	*	*	*	*		*		*	*	*	
High-middle SDI	1990 (95% UI)	664953 (572161 to 841878)	152.5 (132.4 to 191.0)	100950 (87172 to 131573)	25.3 (21.9 to 33.0)	55146 (48232 to 70500)	16.4 (14.2 to 21.2)	1047288 (905993 to 1326208)	263.5 (228.0 to 337.2)	61704 (44675 to 84260)	14.7 (10.6 to 19.8)	985584 (853849 to 1250181)	248.8 (216.6 to 317.5)
	2019 (95% UI)	2398222 (2093974 to 3032510)	259.3 (227.3 to 325.8)	317960 (274781 to 403994)	35.7 (30.4 to 45.1)	114276 (97026 to 142019)	14.8 (12.5 to 18.3)	2025327 (1759184 to 2485485)	235.2 (202.7 to 288.0)	204081 (145478 to 275225)	22.4 (16.0 to 30.2)	1821246 (1566544 to 2223170)	212.7 (182.4 to 260.4)
	Significant?	*	*	*		*		*		*		*	
High SDI	1990 (95% UI)	2529090 (1890053 to 2894921)	571.5 (430.5 to 653.2)	326925 (233497 to 380992)	75.2 (53.9 to 87.9)	99936 (74327 to 116052)	25.7 (19.2 to 30.1)	1807072 (1326671 to 2106492)	426.3 (313.8 to 496.4)	226759 (148240 to 300120)	51.7 (33.9 to 67.9)	1580313 (1169264 to 1855154)	374.6 (278.9 to 438.7)

Region	Year	Prevalence		Incidence		Deaths		DALYs		YLDs		YLLs	
		Number	Rate	Number	Rate	Number	Rate	Number	Rate	Number	Rate	Number	Rate
	2019 (95% UI)	5914063 (5075402 to 8210871)	678.6 (581.4 to 940.4)	696286 (588170 to 994359)	79.7 (67.3 to 113.7)	157989 (131249 to 215133)	17.6 (14.7 to 23.9)	2687711 (2297889 to 3708190)	303.5 (259.1 to 417.3)	477003 (335025 to 703623)	54.6 (38.3 to 80.3)	2210707 (1870801 to 3029625)	248.9 (210.6 to 339.8)
	Significant?	*		*		*		*		*		*	

DALYs: disability-adjusted life years; YLDs: years lived with disability; YLL: years of life lost
