# Supplementary material for: Global and regional quality of care index for prostate cancer: an analysis from the Global Burden of Disease study 1990–2019
Source: Arch Public Health. 2023 Apr 26;81:70. doi: 10.1186/s13690-023-01087-2 (PMC10131390; doi:10.1186/s13690-023-01087-2)
Supplement: Supplementary file 7 — Additional file 7: Supplementary Table S4. Countries with highest QCI changes. [file 13690_2023_1087_MOESM7_ESM.pdf]

Rank	Country	1990 QCI	2019 QCI	QCI change
**1**	Maldives	34.38	76.00	+41.62
**2**	China	39.82	79.17	+39.35
**3**	Equatorial Guinea	0.00	35.67	+35.67
**4**	Bhutan	10.19	44.71	+34.52
**5**	Turkey	48.13	81.22	+33.10
**6**	Bangladesh	10.73	43.23	+32.50
**7**	Peru	45.82	77.59	+31.77
**8**	Bolivia	25.15	56.86	+31.71
**9**	Vietnam	35.59	67.07	+31.48
**10**	Republic of Korea	58.19	89.62	+31.43
