# Supplementary material for: Global and regional quality of care index for prostate cancer: an analysis from the Global Burden of Disease study 1990–2019
Source: Arch Public Health. 2023 Apr 26;81:70. doi: 10.1186/s13690-023-01087-2 (PMC10131390; doi:10.1186/s13690-023-01087-2)
Supplement: Supplementary file 8 — Additional file 8: Supplementary Table S5. Countries with lowest QCI changes. [file 13690_2023_1087_MOESM8_ESM.pdf]

Rank	Country	1990 QCI	2019 QCI	QCI change
**204**	Zimbabwe	28.16	28.53	+0.36
**203**	Tajikistan	41.24	41.77	+0.54
**202**	Dominica	62.52	63.85	+1.32
**201**	Uzbekistan	49.05	53.04	+3.99
**200**	United States of America	94.67	99.71	+5.04
**199**	Saint Vincent and the Grenadines	58.68	64.19	+5.51
**198**	Central African Republic	2.16	7.99	+5.83
**197**	Lithuania	84.39	90.54	+6.15
**196**	New Zealand	91.72	98.04	+6.32
**195**	Monaco	83.80	90.25	+6.45
